# Dendroscope: An interactive viewer for large phylogenetic trees

**DOI:** 10.1186/1471-2105-8-460

**Published:** 2007-11-22

**Authors:** Daniel H Huson, Daniel C Richter, Christian Rausch, Tobias Dezulian, Markus Franz, Regula Rupp

**Affiliations:** 1 Center for Bioinformatics Tübingen (ZBIT), Eberhard-Karls-Universität Tübingen, Sand 14, 72076 Tübingen, Germany

## Abstract

**Background:**

Research in evolution requires software for visualizing and editing phylogenetic trees, for increasingly very large datasets, such as arise in expression analysis or metagenomics, for example. It would be desirable to have a program that provides these services in an effcient and user-friendly way, and that can be easily installed and run on all major operating systems. Although a large number of tree visualization tools are freely available, some as a part of more comprehensive analysis packages, all have drawbacks in one or more domains. They either lack some of the standard tree visualization techniques or basic graphics and editing features, or they are restricted to small trees containing only tens of thousands of taxa. Moreover, many programs are diffcult to install or are not available for all common operating systems.

**Results:**

We have developed a new program, Dendroscope, for the interactive visualization and navigation of phylogenetic trees. The program provides all standard tree visualizations and is optimized to run interactively on trees containing hundreds of thousands of taxa. The program provides tree editing and graphics export capabilities. To support the inspection of large trees, Dendroscope offers a magnification tool. The software is written in Java 1.4 and installers are provided for Linux/Unix, MacOS X and Windows XP.

**Conclusion:**

Dendroscope is a user-friendly program for visualizing and navigating phylogenetic trees, for both small and large datasets.

## Background

Phylogenetic trees are used to represent evolutionary relationships between biological taxa, while taxonomical hierarchies such as the NCBI taxonomy are used to structure the wealth of molecular sequence data. The size of trees under consideration is growing larger and larger.

The Tree of Life project [1], which aims at reconstructing the evolutionary relationship of all living species on earth, now considers more than 11,000 species. The Ribosomal Database Project II provides a hierarchical browser for a collection of approximately 340,000 ribosomal RNA sequences. Recent metagenomic analysis software [2] makes use of the full NCBI taxonomy, which now contains more than 390,000 taxa, to estimate the taxonomical content of a dataset.

Most currently available tree viewers are designed to handle trees containing up to a few thousand nodes. A notable exception is TreeJuxtaposer [3], which was explicitly designed to visualize large trees. While TreeJuxtaposer is the tool of choice for very large datasets (containing hundreds of thousands of taxa), it has limited value as an all-round tree visualization tool, as it only implements one particular tree view (namely the rectangular phylogram, perhaps because this is the only view that is useful for large trees), it lacks basic graphics export capabilities and it does not allow one to save and reopen a modified tree.

## Results and Discussion

**Figure 1:**
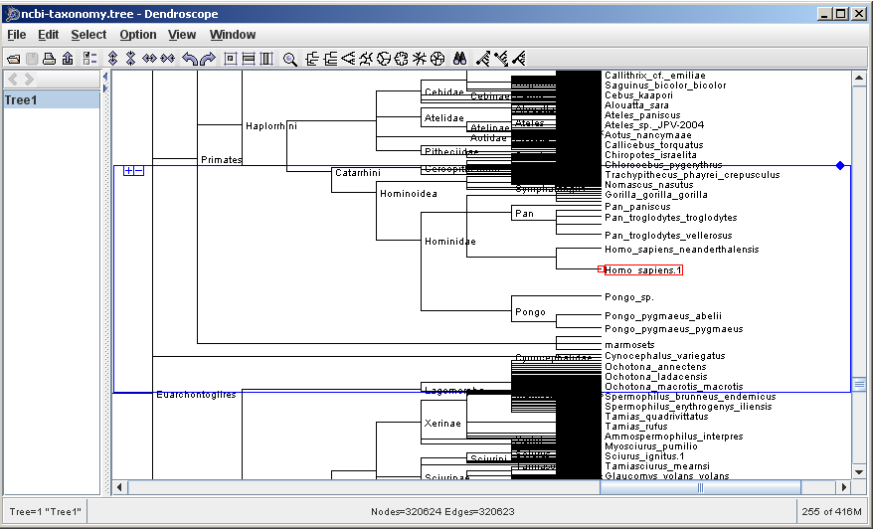
***Homo sapiens *in the NCBI taxonomy**. The placement of *Homo sapiens *and the Hominidae in the NCBI taxonomy, as displayed in Dendroscope using the program's magnifier feature.

Dendroscope is designed as an all-round tree visualization tool that can handle trees with hundred thousands of taxa (see Figure 1). Trees can be read and written in Newick or Nexus format [4], as produced by standard tree reconstruction programs. Additionally, Dendroscope uses its own file format to save and reopen (lists of) trees that have been edited graphically using different colors, line widths and fonts.

**Figure 2:**
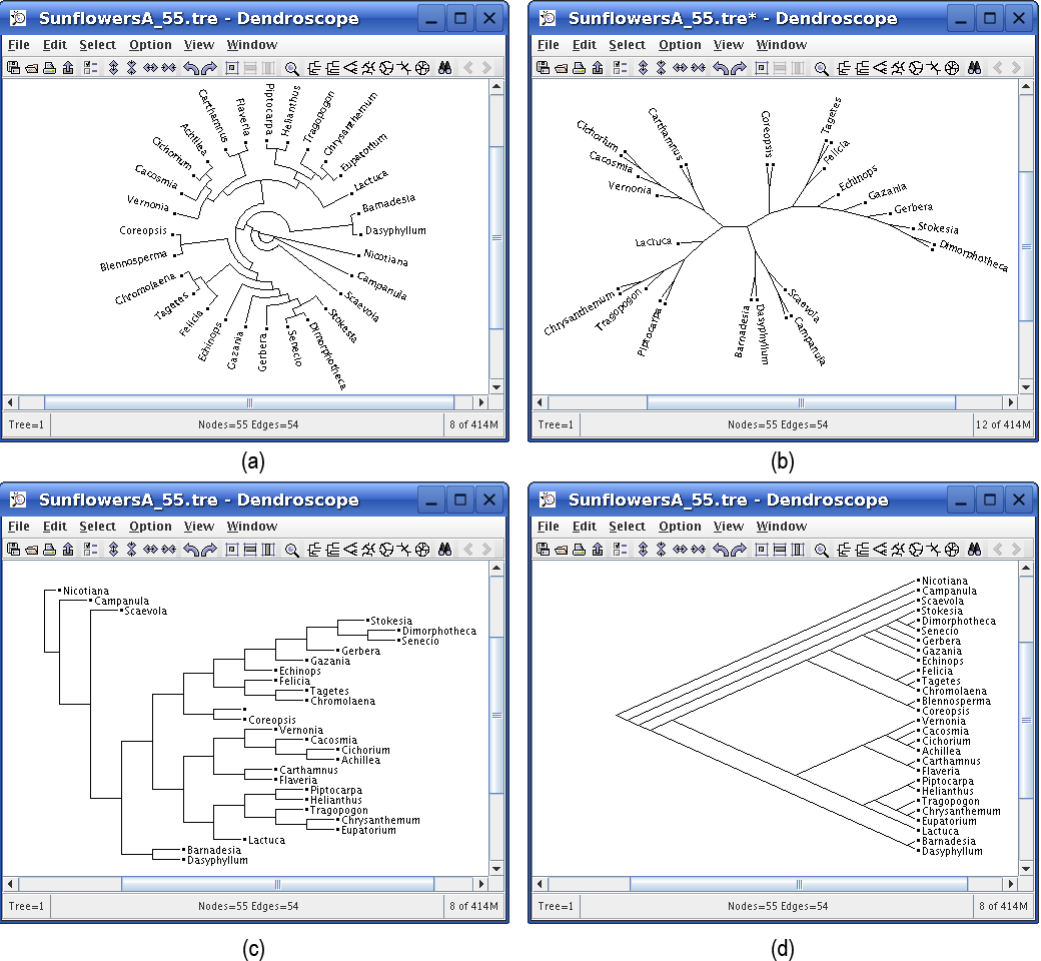
**Four different views of the same dataset**. Four different views for the same dataset of 28 sequences of genera of the daisy family: (a) circular cladogram, (b) radial phylogram, (c) rectangular phylogram, and (d) slanted cladogram.

**Figure 3:**
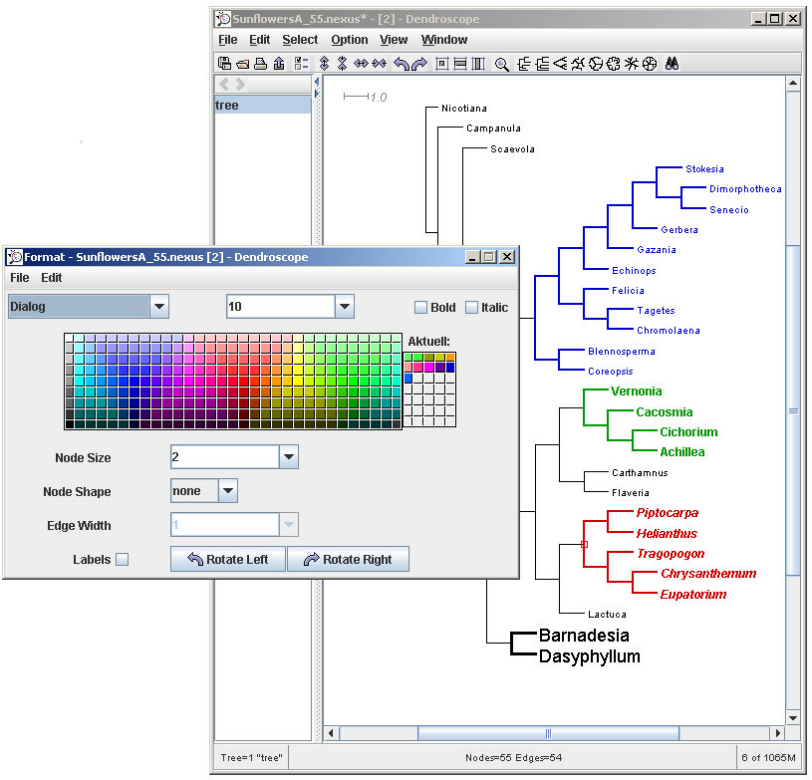
**Formatting nodes and edges of a tree**. Dendroscope provides a dialog box for formatting the nodes and edges of a tree; the example shows a tree drawn as an internal circular cladogram.

A tree can be displayed in a number of views, namely as a circular, radial or rectangular phylogram, as (an internal or external) circular, rectangular or slanted cladogram, or as an unrooted diagram (see Figure 2). The nodes, edges and labels of a tree can be interactively formatted and edited (see Figure 3). Trees can be rerooted and subtrees can be rotated, collapsed, extracted and removed. In the rectangular and slanted views, a horizontal magnifier band can be used to enlarge a part of the tree. In the circular and radial views, a circular magnifier is available, which can also be switched to "magnify all mode", if desired (in which the complete tree is visible under the magnifier). A search tool can be used to find and locate taxa in the tree. All views are exportable as EPS, SVG, PNG, JPEG, GIF and BMP graphic files. Installers are available for Linux/Unix, MacOS X and Windows XP.

### Comparison with other tree viewers

In a survery of existing tree vizualisation and manipulation programs we screened over 40 programs (for extensive lists of such programs, e.g. see [5,6]). In Table 1, we compare Dendroscope to a selection of tree viewing programs which are either widely used or have exceptional features: ATV [7], HyperTree [8], MEGA [9], PHYLIP's [10] drawtree/drawgram, SplitsTree4 [11], TreeView [12], TreeJuxtaposer [3] and TreeDyn [13]. Of the existing programs, only TreeJuxtaposer and PHYLIP's drawtree and drawgram can handle very large trees. PHYLIP's drawtree and drawgram are non-interactive and so are of limited use. TreeJuxtaposer is currently the viewer of choice for large trees. SplitsTree4 and TreeJuxtaposer provide different mechanisms for comparing two or more trees. TreeDyn provides useful features such as scriptability, interoperability with tree databases and especially the possibility to display and manipulate many trees in parallel. Its drawbacks are the limit to trees of only moderate size and the complex user interface.

**Table 1:** Comparison of popular tree viewers. Description of column headers: A: displayable taxa (see Methods section for details), B: search function, C: tree comparison, D: coloring of subtrees, E: editing of labels, F: collapsing of subtrees, G: rerooting, H: rectangular view, I: slanted view, J: radial view, K: circular view, L: graphic export formats

	A	B	C	D	E	F	G	H	I	J	K	L
ATV	2 k	✓				✓	✓	✓				pdf
Dendroscope	350 k	✓		✓	✓	✓	✓	✓	✓	✓	✓	eps, svg, png, jpg, gif, bmp
HyperTree	20 k	✓		✓^1^			✓	✓		✓		-
MEGA	20 k			✓	✓	✓	✓	✓	✓	✓	✓	emf
PHYLIP	1336 k^2^							✓	✓	✓	✓	ps, bmp, pict, pov, fig
SplitsTree4	1 k	✓	✓^3^		✓	✓	✓		✓	✓		eps, svg, png, jpg, gif, bmp
TreeDyn	5 k	✓		✓	✓	✓	✓	✓	✓	✓	✓	ps, svg, png, jpg, gif, etc.
TreeJuxtaposer	1002 k	✓	✓	✓		✓		✓				-
TreeView	2 k^4^			✓^5^	✓^6^	✓	✓	✓	✓	✓		wmf, emf

^1^only single edges

^2^only if "Iterate to improve tree" is set to "no", though trees become illegible as there is no possibility of hiding or magnifying subtrees

^3^using consensus networks

^4^TreeViewX (equivalent to version 0.95 of TreeView): 50 k

^5^only labels

^6^only internal nodes

The system requirements of existing viewers vary: some work only with particular versions of Unix/Linux or MacOS, or they need additional software to be installed. However, all viewers listed in Table 1 run on Linux/Unix, MacOS and Windows, except MEGA, which runs only on Windows.

### Dendroscope at work

Our objective was to build a tree viewer that is able to handle a tree as large as the current version of the NCBI taxonomy. On a standard laptop, Dendroscope performs well on this tree in all rectangular and slanted views. Circular and radial view are less suitable for very large data sets. Figure 1 shows a screenshot of the NCBI taxonomic tree loaded in Dendroscope showing *Homo sapiens *and the Hominidae. Figure 2 demonstrates some of the views provided by the program.

## Conclusion

With Dendroscope, we have developed a new all-round tree viewer that combines all major features found in popular viewers into a single program that can handle large datasets.

## Availability and Requirements

Dendroscope is freely available and can be downloaded from . The software is written in Java 1.4 and installers are provided for Linux/Unix, MacOS X and Windows.

## Methods

### Processing of trees

Since we want to represent very large trees, we need to be able to focus on the crucial parts of the representation to speed up calculations. To this end, we use bounding boxes: to each subtree, we assign a box containing the subtree. The tree is drawn from the root down, and each subtree is drawn only if its bounding box is in the visible region or at least intersects with it. In addition, we compare the height of the bounding box to the number of edges it contains; if we find too many edges in a too small a box, we draw the box as an opaque single element instead of drawing each edge separately. When we want to identify the element (edge or node) at a selected position, we also make use of the bounding boxes: The tree is searched from the root down, leaving out all subtrees whose bounding boxes do not contain the selected position. This reduces the search time from O
 MathType@MTEF@5@5@+=feaafiart1ev1aaatCvAUfKttLearuWrP9MDH5MBPbIqV92AaeXatLxBI9gBaebbnrfifHhDYfgasaacPC6xNi=xH8viVGI8Gi=hEeeu0xXdbba9frFj0xb9qqpG0dXdb9aspeI8k8fiI+fsY=rqGqVepae9pg0db9vqaiVgFr0xfr=xfr=xc9adbaqaaeGacaGaaiaabeqaaeqabiWaaaGcbaWenfgDOvwBHrxAJfwnHbqeg0uy0HwzTfgDPnwy1aaceaGae8NdX=eaaa@3763@(*n*) to O
 MathType@MTEF@5@5@+=feaafiart1ev1aaatCvAUfKttLearuWrP9MDH5MBPbIqV92AaeXatLxBI9gBaebbnrfifHhDYfgasaacPC6xNi=xH8viVGI8Gi=hEeeu0xXdbba9frFj0xb9qqpG0dXdb9aspeI8k8fiI+fsY=rqGqVepae9pg0db9vqaiVgFr0xfr=xfr=xc9adbaqaaeGacaGaaiaabeqaaeqabiWaaaGcbaWenfgDOvwBHrxAJfwnHbqeg0uy0HwzTfgDPnwy1aaceaGae8NdX=eaaa@3763@(*log*(*n*)).

We supply two different magnifiers to let the user easily access inner nodes and taxa: a horizontal magnifier band for rectangular and slanted views, and a circular one for radial tree views. In both cases, a point with distance *d *to the center of the magnifier is mapped to a point with distance f(d)=D/2⋅dd+D/12
 MathType@MTEF@5@5@+=feaafiart1ev1aaatCvAUfKttLearuWrP9MDH5MBPbIqV92AaeXatLxBI9gBaebbnrfifHhDYfgasaacPC6xNi=xH8viVGI8Gi=hEeeu0xXdbba9frFj0xb9qqpG0dXdb9aspeI8k8fiI+fsY=rqGqVepae9pg0db9vqaiVgFr0xfr=xfr=xc9adbaqaaeGacaGaaiaabeqaaeqabiWaaaGcbaGaemOzayMaeiikaGIaemizaqMaeiykaKIaeyypa0JaemiraqKaei4la8IaeGOmaiJaeyyXICDcfa4aaSaaaeaacqWGKbazaeaacqWGKbazcqGHRaWkcqWGebarcqGGVaWlcqaIXaqmcqaIYaGmaaaaaa@3E5F@ from the center, where *D *denotes the diameter or height of the magnifier, as appropriate.

### Test data and system

To estimate the number of displayable taxa for each viewer (see Table 1), we applied the viewer to a list of trees containing increasingly large numbers of taxa: 1 k, 2 k, 5 k, 10 k, 20 k, 50 k, 100 k, 200 k, 334 k, 668 k, 1002 k, 1336 k and 2004 k. In Table 1, we report the maximal size of dataset that could be opened by the viewer, and then loaded and browsed in a reasonable amount of time (less than 90 seconds to open and an interaction response time of less than 15 seconds) on a standard workstation.

## Authors' contributions

All authors participated in the specification and testing of the program. The overall software design is credited to DHH. The program was mainly written by DHH with contributions from TD, MF, CR, DCR and RR. RR worked on the mathematical aspects of the magnification algorithm and contributed to the manuscript. CR and DCR evaluated existing tree viewers, generated test datasets and wrote the main draft of the paper. All authors read and agreed with the final manuscript.
